# Supplementary material for: Identification of photoperiod-induced specific miRNAs in the adrenal glands of Sunite sheep (Ovis aries)
Source: Front Vet Sci. 2022 Jul 22;9:888207. doi: 10.3389/fvets.2022.888207 (PMC9354845; doi:10.3389/fvets.2022.888207)
Supplement: Supplementary file 1 [file Data_Sheet_1.ZIP › Supplementary file/miRDeep2 file.zip!/miRDeep2 file/Novel_Structure/LP42A1/Novel_360.pdf]

Provisional ID	:	NC_019467.2_18043
Score total	:	19.4
Score for star read(s)	:	3.9
Score for read counts	:	12.4
Score for mfe	:	1.6
Score for randfold	:	1.6
Score for cons. seed	:	
Total read count	:	36
Mature read count	:	32
Loop read count	:	0
Star read count	:	4

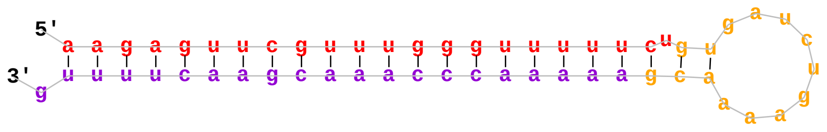

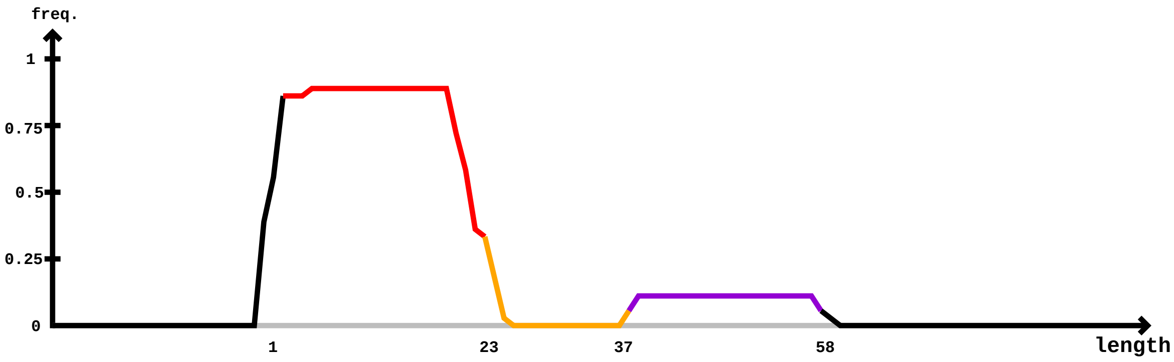


```
                        Mature                     Star
5'- acauguauaaauacauaggucaaagaguucguuuggguuuuucugugaucugaaaacgaaaaacccaaacgaacuuuuggcugacucaauauauauaacauuaacaugucua   -3'   obs
    acauguauaaauacauaggucaaagaguucguuuggguuuuucugugaucugaaaacgaaaaacccaaacgaacuuuuggcugacucaauauauauaacauuaacaugucua         exp
    ..(((((((........((((((.(((((((((((((((((((.((.........))))))))))))))))))))))))))).......))))))).((((....))))...    reads    mm    sample
    ....................caaaAaguucguuuggguuuu.......................................................................    5        1     seq
    ....................caaagaguucguuuggguuuuu......................................................................    1        0     seq
    ....................caaaAaguucguuuggguuuuu......................................................................    7        1     seq
    ....................caaaAaguucguuuggguuuuuc.....................................................................    1        1     seq
    .....................aaaAaguucguuuggguuu........................................................................    6        1     seq
    ......................aaAaguucguuuggguuuuucu....................................................................    11       1     seq
    .........................aguuUguuuggguuuuucugu..................................................................    1        1     seq
    ..........................................................aaaaacccaaacgaacuuuu..................................    1        0     seq
    ..........................................................aaaaacUcaaacgaacuuuug.................................    1        1     seq
    ...........................................................aaaacccaaacgaacuuuu..................................    1        0     seq
    ...........................................................aaaacccaaacgaacuuuuUg................................    1        1     seq
```
